# Predicting lymph node metastasis and recurrence in patients with early stage colorectal cancer

**DOI:** 10.3389/fmed.2022.991785

**Published:** 2022-09-15

**Authors:** Lei Chen, Funing Yang, Zhaoyan Qi, Jiandong Tai

**Affiliations:** ^1^Colorectal and Anal Surgery, General Surgery Center, First Hospital of Jilin University, Changchun, Jilin, China; ^2^Pediatric Outpatient Clinic, First Hospital of Jilin University, Changchun, Jilin, China

**Keywords:** colorectal cancer, tumor budding, lymph node metastasis, predictive nomogram, recurrence-free survival, CDX2, risk stratification

## Abstract

Tumor budding (TB), a powerful, independent predictor of colorectal cancer (CRC), is important for making appropriate treatment decisions. Currently, TB is assessed only using the tumor bud count (TBC). In this study, we aimed to develop a novel prediction model, which includes different TB features, for lymph node metastasis (LNM) and local recurrence in patients with pT1 CRC. Enrolled patients (*n* = 354) were stratified into training and validation cohorts. Independent predictors of LNM and recurrence were identified to generate predictive nomograms that were assessed using the area under the receiver operating characteristic (AUROC) and decision curve analysis (DCA). Seven LNM predictors [gross type, histological grade, lymphovascular invasion (LVI), stroma type, TBC, TB mitosis, and TB CDX2 expression] were identified in the training cohort. LNM, histology grade, LVI, TBC, stroma type, and TB mitosis were independent predictors of recurrence. We constructed an LNM predictive nomogram with a high clinical application value using the DCA. Additionally, a nomogram predicting recurrence-free survival (RFS) was constructed. It presented an AUROC value of 0.944 for the training cohort. These models may assist surgeons in making treatment decisions. In the high-risk group, radical surgery with a postoperative adjuvant chemotherapy was associated with RFS. Postoperative chemotherapy can be better for high-risk patients with pT1 CRC. We showed that TB features besides TBC play important roles in CRC pathogenesis, and our study provides prognostic information to guide the clinical management of patients with early stage CRC.

## Introduction

Owing to recent advances in diagnosis and treatment techniques, endoscopic resection has become the first choice of treatment for early stage colorectal cancer (CRC). However, the optimal management of such excisable tumors is still undefined because of potential metastases; thus, additional surgical resection is necessary to assess nodal status, but the frequency of lymph nodal metastasis (LNM) is relatively low (1). Previous studies have presented guidelines and proposed specific indicators for recommending completion surgery after endoscopic excision to prevent LNM or recurrence (2, 3). However, only approximately 10% (4–6) of patients who were referred for additional surgery based on these guidelines required it. Traditional pathological indicators are not sufficient to identify the need for additional surgery (7). Therefore, reliable criteria to assess patients requiring surgery are crucial.

Besides the resection margin, other promising indicators for additional surgical intervention include the tumor grade, lymphovascular invasion (LVI), and tumor budding (TB). The latter is characterized by the dissociation of small tumor complexes containing up to four cells that “bud” into the intratumoral or peritumoral stroma. TB is associated with a high risk of LNM in patients with pT1 CRC. Consequently, patients with pT1 CRC marked by prominent TB may benefit from additional surgical resection (8, 9). TB assessed in pre-operative biopsies could predict tumor regression for neo-adjuvant chemotherapy (10). Furthermore, high-level TB is a high-risk factor for patients with stage II CRC, and thus can warrant the consideration of adjuvant chemotherapy. Therefore, further studies are needed to determine whether TB assessments can help guide high-risk patients with pT1 CRC to undergo postoperative adjuvant chemotherapy to improve outcomes. The International Tumor Budding Consensus Conference (ITBCC) guidelines provide a standardized counting system for routine reporting. However, several factors should be considered when using the ITBCC TB scoring system in routine practice. First, the current ITBCC three-tier system (Bd1, Bd2, and Bd3) is the same for all stages of CRC. Both Bd2 and Bd3 are considered high-risk factors for LNM in pT1 CRC, whereas in stage II CRCs, only Bd3 is a risk factor for poor survival. Second, reporting of the absolute number of tumor buds is recommended, although inconsistency in tumor bud counts among pathologists may lead to differences in clinical management. Finally, the current TB assessment system focuses only on the tumor bud count and does not account for other features of TB, including structure, location, cell atypia, stroma type, tumor bud cell mitosis, and the immunohistochemical phenotype of the tumor bud cells. Including these other parameters in predictive models could improve the risk stratification power and prognostic value of TB and its various features.

In this retrospective study, we aimed to analyze the clinicopathologic characteristics to evaluate the risk stratification utility of TB features in early stage CRC. Furthermore, we developed a novel nomogram, including different characteristics of TB, to guide adjuvant chemotherapy in patients with early stage CRC. This approach could be combined with traditional clinicopathological indicators to assist surgeons in choosing the most suitable operation for patients with early stage CRC.

## Materials and methods

### Patients

This retrospective study included 354 consecutive patients who were pathologically diagnosed with pT1 CRC and who underwent radical surgery between January 2010 and December 2018 in the First Hospital of Jilin University (Changchun, China). Sixty patients received chemotherapy with fluorouracil plus oxaliplatin after surgery. We excluded patients who (i) underwent only endoscopic excision; (ii) with missing follow-up data; (iii) with specific histological subtypes of adenocarcinomas, such as poorly cohesive carcinoma, signet-ring cell carcinoma, micropapillary adenocarcinoma, mucinous adenocarcinoma, and medullary adenocarcinoma; and (iv) with more complicated or advanced CRC, higher than stage T1. The study protocol was approved by the institutional ethics committee of Jilin University First Hospital. The need for written informed consent was waived because of the retrospective nature of the study.

### Histology

Hematoxylin and eosin-stained slides were reviewed by two pathologists. All slides were reviewed in a double-blinded manner, without knowledge of the corresponding pathological diagnoses. The initial clinical and pathological stages of the disease in all patients were revised according to the American Joint Committee on Cancer staging system (eighth edition). Histological type and grade were defined according to the latest World Health Organization classification system. In all specimens, the following histological features were evaluated: the LVI, predominant structure of tumor bud (cluster or single-cell), predominant location of TB (peritumoral or intratumoral budding), and tumor bud cell atypia (non-specific or anaplasia-like; anaplasia was defined as any × 400 magnification field with ≥ 3 nuclei with diameters equal to or greater than 5 lymphocyte nuclei) (11), stroma type (inflammatory, fibrotic, or myxoid; the predominant feature was recorded), mitosis in tumor bud cells, and tumor bud count. TB was defined as the dissociation of small tumor complexes containing more than five cells that “budded” into the intratumoral or peritumoral stroma. TB was scored by two independent pathologists according to the ITBCC guidelines (12). Hematoxylin and eosin-stained sections were evaluated at medium magnification (× 10) to determine the densest area of TB at the invasive tumor front (“hotspot”). To reduce interobserver variability, TB features were independently evaluated by two single-blinded pathologists. The final classification of TB features was determined based on agreement among at least two pathologists.

### Immunohistochemistry

Immunohistochemistry was performed as described previously (13). Tissue sections were stained using the following primary antibodies: rabbit monoclonal CDX2 (EP25; Zhongshan Golden Bridge Biotechnology LLC, Beijing, China; ready-to-use); Ki-67 (30-9; Ventana, Tucson, AZ, United States; ready-to-use), epidermal growth factor receptor (EGFR; EP22; Zhongshan Golden Bridge Biotechnology LLC; ready-to-use), p53 (4A4 + UMAB4; Zhongshan Golden Bridge Biotechnology LLC; ready-to-use), BRAF V600E (VE1; Ventana; ready-to-use), and microsatellite instability (MSI) proteins, including MLH1 (ES05), PMS2 (EP51), MSH2 (RED2), and MSH6 (EP49) (Zhongshan Golden Bridge Biotechnology LLC; ready-to-use).

CDX2 and EGFR immunohistochemical staining were performed as described previously (14). The extent of tumor bed cell staining (0–100%) and the staining intensity (0, negative; 1, weak brown; 2, brown; and 3, dark brown) were evaluated. The final scores were defined as the product of the extent and intensity scores. Next, each case was scored as high or low, using the median final score as the cut-off point for the following test. The immunohistochemical staining patterns of p53 were classified into two subgroups: (a) wild-type pattern, indicated by scattered nuclear staining in tumor cells, and (b) mutant-type pattern, in which the majority of tumor cells (> 60%) showed diffuse strong nuclear positivity or were completely devoid of any staining. Only staining for the expression of cytoplasmic BRAF V600E was considered positive. The MSI status was classified into two subgroups: (a) MSI-high, if any one of the four mismatch repair proteins (MLH1, PMS2, MSH2, and MSH6) was nuclear negative in all tumor cells, but positive in internal controls; and (b) MSI-low, if all four mismatch repair proteins were positive in cancer cells. The p53- and BRAF-staining patterns and MSI status were reported by two single-blinded observers.

### Statistical analysis

The clinicopathological findings of the CRC specimens were compared using the chi-square or Fisher’s exact test for categorical variables. The non-parametric Mann–Whitney *U* test was used to analyze age, Ki67 labeling index, and TBC datasets because of their non-normal distribution.

Multivariate logistic and Cox regression analyses were used to identify significant independent factors for predicting LNM or recurrence-free survival (RFS). Variables with *P* < 0.1 in the univariate analysis were included in the multivariate analysis model. The RFS of the patients was analyzed using the Kaplan–Meier method and log-rank test. *P* values were obtained using two-tailed statistical analyses, and the significance level was set at 5% (*P* < 0.05). R software (version 4.1.0^1^) was used for all statistical analyses. The R statistical packages “rms,” “barplot,” “survival,” “Hmisc,” “MASS,” and “pROC” were used to plot the distribution of risk scores and recurrence or distant metastasis, plot calibration, generate receiver operating characteristic curve, build a nomogram, and draw Kaplan–Meier curves. The package “rmda” was used to draw the decision curve analysis (DCA) curves, and “forestplot” was used to draw the forest plot.

## Results

### Demographic and clinicopathological findings

The baseline clinicopathological characteristics of the participants (*n* = 354) are summarized in Table 1. LNM was present in 49 (13.8%) patients (mean age ± standard deviation = 65.2 ± 10.3 years; range = 30–91 years). Recurrence was observed in 38 (10.7%) patients, and the follow-up period was 37.4 ± 16.3 months (range = 14.2–59.9 months).

**TABLE 1:** Demographics of surgery of 354 patients with pT1 CRC who underwent surgical resection.

Variable		All patients
Age (years)*		65.2 ± 10.3 [30–91]
Sex	Female	131 (37.0%)
	Male	223 (63.0%)
LNM	Absent	305 (86.2%)
	Present	49 (13.8%)
Gross tumor type	Non-pedunculated	161 (45.5%)
	Pedunculated	193 (54.5%)
*TP53*	Wild-type	27 (7.6%)
	Mutant-type	156 (44.1%)
	-	171 (48.3%)
*MSI*	MSI-high	11 (3.1%)
	MSI-low	172 (48.6%)
	-	171 (48.3%)
*BRAF*	Absent	182 (51.4%)
	Present	1 (0.3%)
	-	171 (48.3%)
Ki67 (%)*		78.7 ± 13.2 [5.0–95.0]
Histology grade	Low-grade	339 (95.8%)
	High-grade	15 (4.2%)
Lymph-vascular invasion	Absent	315 (89.0%)
	Present	39 (11.0%)
TB construction	Cluster	201 (56.8%)
	Single	153 (43.2%)
TB location	ITB	118 (33.4%)
	PTB	236 (66.7%)
TB atypia	Non-specific	311 (87.9%)
	Anaplasia-like	43 (12.1%)
TB stroma	Inflammation	100 (28.3%)
	Fibrosis	192 (54.2%)
	Myxoid	62 (17.5%)
TB mitosis	Absent	298 (84.2%)
	Present	56 (15.8%)
TB quantity*		10.7 ± 3.7 [0.0–18.0]
TB CDX2 status	Negative	87 (24.6%)
	Positive	267 (75.4%)
TB EGFR status	Negative	50 (14.1%)
	Positive	304 (85.9%)
Recurrence	Absent	316 (89.3%)
	Present	38 (10.7%)

*Data are mean ± standard deviation. MSI, microsatellite instability; TB, tumor budding.

### Evaluation and validation of the lymph node metastasis predictive nomogram

**FIGURE 1:**
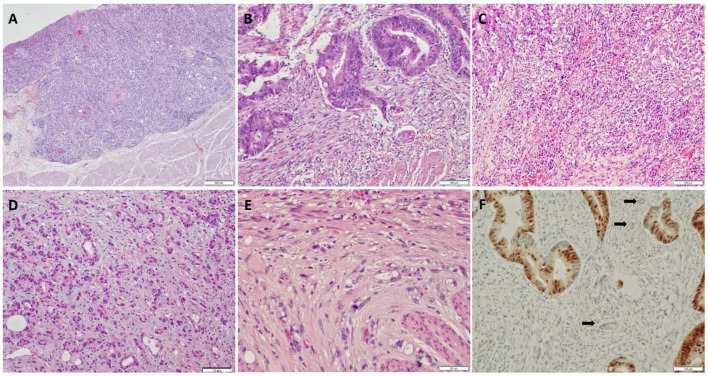
Histological and immunohistochemical features of pT1 colorectal cancer (CRC). **(A)** High histology grade; **(B)** lymph-vascular invasion observed in a biopsy specimen; **(C)** inflammatory stroma surrounding tumor budding (TB); **(D)** myxoid stroma surrounding TB; **(E)** mitosis present in TB; **(F)** CDX2 expression in tumor cells, while loss of expression in TB.

**FIGURE 2:**
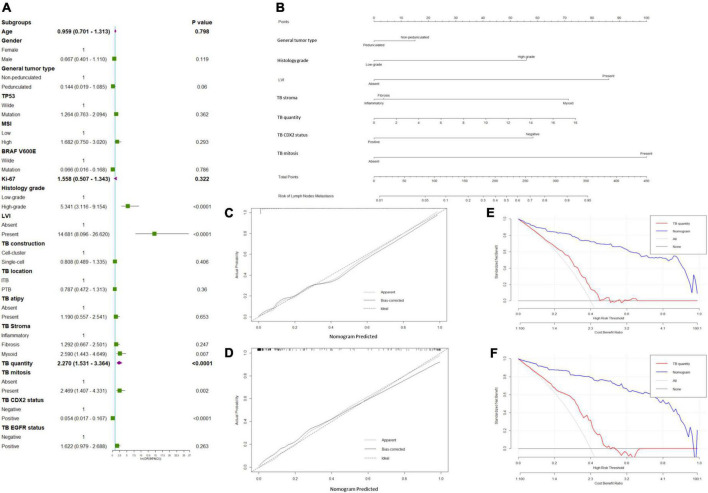
Predicted model of lymph node metastasis (LNM). **(A)** Forest plots to decipher the risk factors associated with LNM identified in the univariate logistic regression analysis; **(B)** newly developed nomogram for predicting LNM in patients with pT1 CRC. The calibration curve for predicting LNM of pT1 CRCs in the **(C)** training and **(D)** validation cohorts. Decision curve analysis of the nomogram and TB quantity alone for predicting LNM in patients with pT1 CRC in the **(E)** training cohort and **(F)** validation cohort.

In total, 354 patients were included and randomly allocated to a training cohort (*n* = 234) and validation cohort (*n* = 120) at a ratio of approximately 2–1 based on the data splitting approach. Based on the univariate logistic regression analysis results of the training cohort, seven factors, namely general tumor type, histology grade (Figure 1A), LVI (Figure 1B), tumor bud stroma (Figures 1C,D), tumor bud count, tumor bud cell mitosis (Figure 1E), and CDX2 expression (Figure 1F), were linked to the LNM status (Figure 2A).

General tumor type [pedunculated vs. non-pedunculated; odds ratio (OR) = 0.641; 95% confidence interval (CI) = 0.098–4.185], histological grade (high-grade vs. low-grade; OR = 5.561; 95% CI = 1.933–16.003), LVI (present vs. absent; OR = 34.194; 95% CI = 9.511–122.930), tumor bud cell stroma type (myxoid vs. inflammatory; OR = 6.746; 95% CI = 1.831–24.851), tumor bud count (high vs. low; OR = 63.429; 95% CI = 14.623–275.130), TB mitosis (present vs. absent; OR = 2.770; 95% CI = 0.643–11.925), and TB CDX2 expression status (negative vs. positive; OR = 15.919; 95% CI = 4.259–59.494) were independent predictors of recurrence in the multivariate analyses (Table 2 and Figure 2B).

**TABLE 2:** Multivariate logistic regression analysis of lymph node metastasis.

	Training cohort		Validation cohort	
	(*n* = 234)		(*n* = 120)	
	OR (95% CI)	*P-value*	OR (95% CI)	*P-value*
**Gross tumor type**				
Non-pedunculated	1.000		1.000	
Pedunculated	0.641 (0.098–4.185)	0.477	0.826 (0.124–4.079)	0.772
**Histology grade**				
Low-grade	1.000		1.000	
High-grade	5.561 (1.933–16.003)	0.002	4.403 (1.046–18.520)	0.043
**LVI**				
Absent	1.000		1.000	
Present	34.194 (9.511–122.930)	0.004	11.156 (2.186–56.912)	0.003
**TB stroma**				
Inflammation	1.000		1.000	
Fibrosis	1.667 (1.278–9.451)	0.527	1.206 (1.1412–6.216)	0.087
Myxoid	6.746 (1.831–24.851)	0.032	4.303 (1.945–15.933)	0.022
**TB mitosis**				
Absent	1.000		1.000	
Present	2.770 (0.643–11.925)	0.171	1.013 (0.926–2.618)	0.568
TB quantity	63.429 (14.623–275.130)	0.001	28.952 (4.010–208.990)	0.008
**TB CDX2 status**				
Negative	15.919 (4.259–59.494)	0.021	17.350 (7.689–25.778)	0.003
Positive	1.000		1.000	

CI, confidence interval; OR, odds ratio; MSI, microsatellite instability; LVI, lymphovascular invasion; TB, tumor budding.

The calibration curve of the LNM nomogram was highly consistent with the standard curve, indicating the high reliability of the predictive ability of the nomogram (Figures 2C,D). The DCA curves for the developed LNM nomogram (Figure 2E) and tumor bud count (Figure 2F) in the training and validation cohorts indicated that the DCA of the predictive nomogram had higher net benefits than the tumor bud count, indicating higher clinical application value.

### Evaluation and validation of the recurrence-free survival prediction nomogram

**FIGURE 3:**
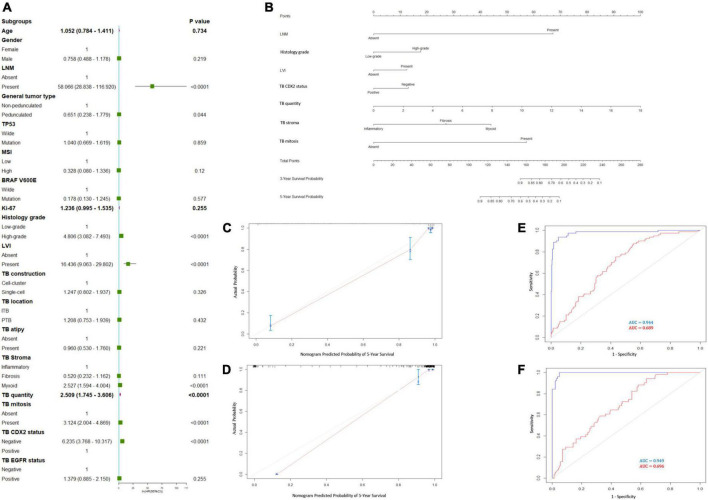
Prediction model for recurrence-free survival (RFS). **(A)** Forest plots to decipher the risk factors associated with RFS identified in univariate Cox regression analysis; **(B)** RFS predictive nomogram. The calibration curve of postoperative RFS in patients with pT1 CRC in the **(C)** training cohort and **(D)** validation cohort. Predictive accuracy of RFS-nomogram in the **(E)** training cohort and **(F)** validation cohort.

Cox univariate and multivariate regression analyses were performed in the training cohort to identify the variables for building the RFS predictive nomogram. RFS was significantly associated with LNM, general tumor type, histology grade, LVI, stroma type, tumor bud count, tumor bud cell mitosis, and CDX2 expression status (Figure 3A). In the multivariate Cox proportional hazards model, LNM, histology grade, LVI, TBC, stroma type, and TB mitosis were independent predictors of local recurrence (Table 3). These variables were used to build a predictive nomogram for RFS (Figure 3B). Calibration curves based on the six variables are shown in Figures 3C,D. There was a positive agreement between the nomogram-predicted and actual probabilities of 5-year RFS in the training and validation cohorts, respectively. The predictive ability of the RFS nomogram was evaluated by analyzing the area under the ROC (AUROC). The nomograms displayed discriminatory power in predicting the postoperative RFS in the training cohort. The C-indices of the RFS nomogram and tumor bud count were 0.944 (95% CI = 0.934–0.952) and 0.689 (95% CI = 0.642–0.736), respectively (Figures 3E,F).

**TABLE 3:** Multivariate COX regression analysis of recurrence.

	Training cohort		Validation cohort	
	(*n* = 234)		(*n* = 120)	
	HR (95% CI)	*P-value*	HR (95% CI)	*P-value*
**LNM**				
Absent	1.000		1.000	
Present	32.292 (14.401–72.407)	< 0.0001	16.331 (6.007–58.260)	< 0.0001
**Gross tumor type**				
Non-pedunculated	1.000		1.000	
Pedunculated	0.733 (0.171–2.153)	0.337	0.891 (0.432–3.014)	0.547
**Histology grade**				
Low-grade	1.000		1.000	
High-grade	1.622 (1.002–2.627)	0.049	1.548 (0.771–3.107)	0.218
**LVI**				
Absent	1.000		1.000	
Present	2.686 (1.162–6.208)	0.021	2.958 (1.160–7.541)	0.023
**TB stroma**				
Inflammation	1.000		1.000	
Fibrosis	1.256 (0.449–1.632)	0.551	1.192 (0.312–1.880)	0.715
Myxoid	1.719 (0.264–1.955)	0.001	1.280 (0.950–2.819)	0.151
**TB mitosis**				
Absent	1.000		1.000	
Present	1.022 (0.540–1.933)	< 0.0001	1.567 (0.615–2.673)	0.094
TB quantity	1.703 (1.055–2.750)	0.029	1.563 (0.838–2.914)	0.020
**TB CDX2 status**				
Negative	0.935 (0.520–1.679)	0.216	1.789 (0.711–4.502)	0.799
Positive	1.000		1.000	

Statistical analyses were conducted using log-rank tests and a Cox proportional hazards model. CI, confidence interval; LVI, lymphovascular invasion; RFS, recurrence-free survival; TB, tumor budding.

**FIGURE 4:**
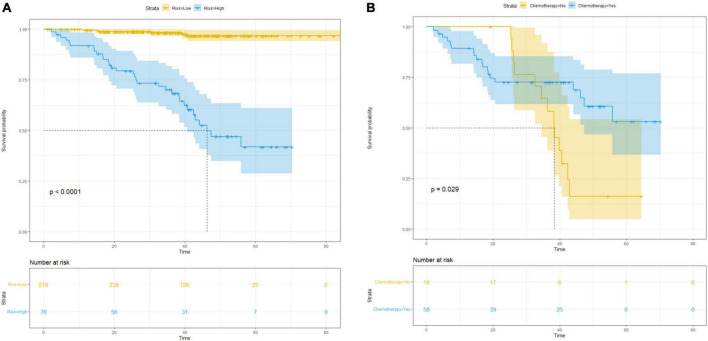
Survival curves for subgroup analysis in patients with different risk of postsurgical recurrence stratified by nomogram score. **(A)** Kaplan–Meier survival curves for RFS according to the risk status in all patients; **(B)** RFS according to different therapy in high-risk cohort.

Based on the nomogram score, patients were stratified into low- (score ≤ 160) and high-risk (score > 160) for recurrence and mortality, respectively. We used Kaplan–Meier curves and the log-rank test to analyze RFS in patients with pT1 CRC after stratification (low-risk vs. high-risk) using the nomogram (*p* < 0.001; Figure 4A). In the high-risk group, patients who only underwent radical surgery had a lower RFS (*p* = 0.029; Figure 4B), compared to that of patients who underwent radical surgery and a postoperative chemotherapy.

## Discussion

Although the traditional TNM staging system remains essential for risk stratification in patients with CRC, the heterogeneity in survival rates within the same stages indicates the need for additional prognostic biomarkers. Furthermore, the invasive front morphology may be more representative of the biological behavior of the tumor than the primary tumor core morphology (15). Our study demonstrated that the morphology and immunohistochemical features of TB in cases of early stage CRC were predictive of tumor progression and local recurrence. Among the various features, non-pedunculated gross type, high histological grade, LVI, myxoid-type tumor bud stroma, high tumor bud count, TB mitosis, and loss of CDX2 expression were independent predictors of LNM, whereas LNM, histology grade, LVI, TBC, stroma type, and TB mitosis were independent predictors of local recurrence in patients with pT1 CRC.

TB is a morphological characteristic that reflects the high aggressiveness of tumors at the invasion margin. A previous study revealed that high-level TB correlated with mutated KRAS or MSS/pMMR (16). Furthermore, patients with CRC who have a KRAS mutation and MSS/pMMR tumor were part of a group with the poorest prognosis (17). It is defined as a morphologic surrogate of epithelial-mesenchymal transition (EMT), a mechanism through which tumor cells acquire motility and invasiveness (18). EMT facilitates the detachment of cancer cells from the tumor mass and their subsequent infiltration in the extracellular matrix as single cells or small clusters (1, 9). TB has a strong risk stratification utility, so much so that the ITBCC system can reliably predict the prognosis of patients with CRC based on tumor bud count alone (19). However, not all patients with a high tumor bud count will have a poor prognosis; thus, further investigation of the characteristics of TB could improve its value for risk stratification. Our study is the first to improve the current ITBCC system by exploring various features of TB to predict LNM in patients with pT1 CRC, rather than limiting the input parameters to the tumor bud count. Moreover, the nomograms that we developed showed stronger discriminative ability than tumor bud count in predicting LNM or local recurrence. Thus, our easy-to-use predictive nomograms could be useful tools to quantify the probability of RFS.

A high histological grade has also been shown to be associated with aggressive tumor biology and to be of prognostic significance; it has been associated with various informative tumor parameters in human malignancies (20–25). Our results agree with the findings of previous studies on non-CRCs and indicate that a high histological grade is of prognostic value for pT1 CRC. Additionally, we focused on the presence of atypia of tumor bud cells, but it was not of statistical significance; it was mainly present in the tumor center. Furthermore, tumor bud cell mitosis was observed in 15.8% of the cases in the present study. Mitosis results from rapid cell proliferation and is correlated with tumor proliferation and invasiveness; it is associated with adverse clinical outcomes in other cancer types (26), although its prognostic value in CRC has not yet been defined. A previous study revealed that a decrease in mitosis is associated with high-level TB (27), which may represent the potential decrease in the mitosis of tumor bud cells because of fibroblastic cells around TB during the EMT process in an effort to slow down tumor invasion. Thus, mitosis in TB may reflect tumor biology and may provide valuable prognostic information.

Unlike the tumor core, the environment at the tumor front is not static; inflammatory, fibrotic, and myxoid stroma are histologic features representing snapshots of the dynamic process of extracellular matrix remodeling. The immature or myxoid stroma desmoplastic reaction has been recognized as an independent prognostic predictor in CRC (28). This feature, however, has not been routinely adopted in pathology reports for clinical care. Furthermore, myxoid stroma is associated with the absence of tumor-infiltrating lymphocytes in CRC (29), which enhance tumor immune escape. Some studies have demonstrated that the immature myxoid stroma is associated with a high degree of tumor budding (30). The myxoid stroma surrounding the tumor buds that appear at the tumor front is regarded as an immature stroma with a high potential to disseminate and metastasize (31). Consistent with the findings of a previous study (32), in the current study, myxoid stroma was significantly associated with the presence of LNM and local recurrence in patients with pT1 CRC.

In addition to the histological characteristics of TB, we also investigated the immunophenotype of TB. We observed a loss of CDX2 expression in 6.6% of tumor buds, which differed from the tumor core. Notably, CDX2 inhibits EMT and metastasis of CRC by regulating Snail and β-catenin expression (33). CDX2, an intestine-specific transcription factor, has been strongly implicated in the development of the intestinal mucosa (34). Emerging evidence suggests the crucial role of CDX2 as a tumor suppressor during colorectal carcinogenesis. CDX2 expression is inversely associated with tumor grade in CRC (35, 36). Consistent with the findings of a previous study, the downregulation of CDX2 expression was associated with LNM in patients with pT1 CRC. The lack of CDX2 expression in tumor buds may indicate that they are in a state of EMT; thus, it could predict poor prognosis in patients with CRC. Moreover, because these features are associated with LNM, they can be applied to endoscopic biopsy specimens to better predict tumor progression behavior. To the best of our knowledge, these findings have not been previously reported. Additional studies with larger cohorts are required to validate the prognostic implications of the histological and immunohistochemical features of TB that may help predict the prognosis or occurrence of LNM.

Our novel nomogram can effectively stratify the recurrence risk in pT1 CRC patients, and the KM survival curve shows that the RFS of high-risk patients is much shorter than that of the low-risk. Furthermore, our data revealed that adjuvant chemotherapy is necessary in the high-risk group. Therefore, adjuvant chemotherapy is recommended for high-risk patients even if they do not have LNM. Our method could be applied to determine risk stratification strategies for patients with pT1 CRC; for example, to identify low-risk patients to avoid unnecessary additional treatment, while identifying high-risk patients to enable timely and effective treatment. Low-risk patients could extend their follow-up periods, improving their quality of life and reducing postoperative complications and financial costs. However, this study had some limitations. First, the statistical power was limited because this was a single-center retrospective study. Second, owing to the retrospective study design, potential selection biases could not be ruled out. Finally, although the study focused on identifying the most significant predictors of LNM and local recurrence, it is unclear whether these findings can be generalized.

In conclusion, tumor bud count and other features of TB are associated with LNM and poor prognosis in patients with pT1CRC. Although tumor bud count, stroma type, and the CDX2 expression status in tumor buds were identified as risk factors for LNM, only tumor bud count was significantly correlated with local recurrence in patients with pT1 CRC. Thus, these features of TB should be incorporated into the routine evaluation of CRC, as they may provide valuable information to guide clinical therapy. Additional studies in a multi-institutional setting are needed to confirm these findings.

## Data availability statement

The original contributions presented in this study are included in the article/supplementary material, further inquiries can be directed to the corresponding author.

## Author contributions

LC and JT performed the material preparation and data collection and analysis. LC wrote the first draft of the manuscript. All authors contributed to the article and approved the submitted version.

## Footnotes

1
www.r-project.org
